# Characterising the behaviours in most severe and least severe emotional outbursts in young people

**DOI:** 10.1038/s41598-024-52732-x

**Published:** 2024-02-05

**Authors:** Benjamin Daniel Kitchen Shenton, Justin Cheuk Yin Chung, Kate Anne Woodcock

**Affiliations:** 1 School of Psychology, University of Birmingham, Birmingham, B15 2SA UK; 2 Centre for Applied Psychology, Institute for Mental Health and Centre for Development Science, School of Psychology, University of Birmingham, Birmingham, UK

**Keywords:** Human behaviour, Social behaviour

## Abstract

Emotional outbursts are displays of intense, challenging behaviour and are prevalent in individuals with neurodevelopmental disorders. Outbursts present a danger to individuals and their carers and are cited as reasons for referral to mental health services. However, it is currently unclear how the characteristics of outbursts may determine their severity. Carers (n = 214) of individuals aged between 6 and 25 and experiencing outbursts at least once per month completed the Emotional Outburst Questionnaire. Questionnaire items were used to compare behaviours observed in most severe and least severe outbursts through quantitative and content analyses of open ended data. Signs of physiological arousal and aggression were seen significantly more in most severe outbursts compared to least severe outbursts. Least severe outbursts were seen more frequently, but most severe outbursts were reported to have a longer duration, be at a higher intensity, and have a longer recovery time. Additionally, associations were found between reduced eye contact and most severe outbursts, as well as expression of suicidal ideation and most severe outbursts. Certain behaviours, notably forms of aggression and physiological arousal, are associated with most severe outbursts. Findings of this study may allow future work examining cross-disorder differences in outbursts to inform targeted interventions aiming to reduce outburst severity and impact. Additionally, identification of such outburst characteristics could aid in measurement of outburst severity, which would allow for more reliable and valid studies on outburst interventions.

## Introduction

Emotional outbursts are intense explosions of challenging behaviours and are often associated with aggression, crying and heightened emotions^1–3^. There is no established definition for outbursts^4^. In the literature emotional outbursts can be referred to as meltdowns, temper outbursts or tantrums^5,6^. Outbursts are prevalent in individuals with neurodevelopmental disorders such as autism spectrum disorder (ASD), intellectual disability and Prader–Willi syndrome (PWS)^6,7^. Outbursts negatively impact upon the individual and their carers and families; increases in symptom severity have been correlated with increased stress and depressive symptoms in their parents and carers^8^. There is a physical danger to the individual and others around them from self-injurious behaviours and aggressive behaviours towards others during outbursts^4^. A study investigating autistic individuals’ experiences of outbursts found that during an outburst they may feel psychologically and physicaly overwhelmed due to feeling not in control of their actions, and may go on to feel further distress afterwards because of remorse for aggressive actions^9^ . Despite the prevalence of emotional outbursts, and their impact on both the individual and those around them, effective interventions targeting outbursts are lacking^4,10^.

Studies have characterised the individual behaviours that make up an outburst in individuals with different diagnoses, such as ASD and PWS^5,11^. Though these studies look at one diagnosis in isolation, when comparing across diagnoses it appears that the behaviours and characteristics that make up an emotional outburst are broadly similar and outbursts can therefore be considered transdiagnostically^10^.

Although there is low between-group variability, our pilot work shows that there is within-individual variability that has not been accounted for by previous studies. Specifically, caregivers can differentiate between the most severe and least severe outbursts displayed by the individual they care for. A small number of studies have identified outburst component behaviours as being more or less demanding or severe^12,13^. Behaviours considered to be more severe included self-injurious behaviours that resulted in harm to the individual or others, or behaviours that required physical intervention from more than one person. However, even if these different behaviours ultimately contribute to differences in outburst severity, when studies only investigate behaviours seen in the typical outburst or collate behaviours from all outbursts, as the above have done, variability of outburst severity within the same individual cannot be identified or measured^5,14^.

Identifying variability in severity of outbursts within the same individual is critical when one begins to think about developing interventions for emotional outbursts and measuring their efficacy. Many intervention studies simply measure occurrence of emotional outbursts as a single numerical value^15,16^. But such measures are not sensitive to improvements in outburst severity that may be mediated by the interventions, which can be an important intervention goal for caregivers^17^. Thus, lacking measures that accurately index changes in outburst severity, intervention development will remain limited by inadequate outcome measures that cannot capture critical change^4,18^.

If an individual experiences behaviour normally associated with more severe outbursts in their least severe outbursts, it might be a sign that their outburst profile is more severe overall, meaning they may require a different level of support. Additionally, since there can be variability in outburst severity within an individual, they might require different levels or forms of intervention for different episodes of outbursts. Thus, knowing the characteristics that typically distinguish outburst severity, and assessing an individual's outburst behaviour with reference to these, could help those supporting young people to select the most appropriate interventions in a more personalised manner. Such an approach to ongoing assessment to inform intervention is in line with current UK guidelines, which recommend that assessment of behaviours in this context should be flexible and continued^19^. Additionally, personalised interventions could yield more benefits than a ‘one-size-fits-all’ approach.

In the present study, we apply the Emotional Outburst Questionnaire, which has been developed for measuring the nature and context of outbursts in detail, and has been validated cross-culturally in terms of outburst contexts^10,20^. Importantly, the questionnaire includes profiling the characteristics of most and least severe outburst types in the same individual.

This study aims to characterise the most and least severe outbursts experienced by young people with neurodevelopmental disorders, including identifying which behaviours are associated with most and least severe outbursts.

## Methods

### Participants

This paper forms part of a larger cross-sectional study investigating emotional outbursts in young people with neurodevelopmental disorders^10^. The current sample is a sub-sample of the wider study. The inclusion criteria for the wider study were that participants must be caring for an individual who is aged between 6 and 25 and experiencing emotional outbursts at least once a month. 268 participants were recruited from support groups in the UK, Ireland, Australia, and North America. A distinction was not made in terms of the caregiver’s relationship with the young person, though most participants were recruited through support groups for families of young people with neurodevelopmental disorders.

The original cohort of participants were asked if the outbursts of the individual they cared for varied. 214 participants answered yes and were included in the present study. The mean age of the individuals the participants cared for was 13.1 years (SD = 5.1, minimum = 6.1, maximum = 25.9). 131 were male, 82 were female, and 1 was non-binary. Appendix A in supplementary materials lists the diagnoses given by five participants or more.

### Procedure

Ethical approval was obtained from the Science, Technology, Engineering and Mathematics Ethical Review Committee at the University of Birmingham. The approval number is ERN_18-1885. All methods were performed in accordance with the relevant guidelines and regulations. Additionally, informed consent was obtained from all participants and/or their legal guardian(s). Participants completed the Emotional Outburst Questionnaire (EOQ), the Social Communication Questionnaire (SCQ) and a demographics questionnaire online.

The focus of this paper is on sections 1 and 2 of the EOQ which can be viewed in full in the supplementary materials. The subsequent sections are the focus of different studies by the research team. The questions that form sections 1 and 2 are detailed in Table 1. In section 1 participants are asked to consider questions in relation to the individual’s most severe outbursts, and in section 2 to consider the same set of questions in relation to least severe outbursts. Caregivers are asked to consider the questions in relation to behaviours that have occurred over the previous one month.

**Table 1:** Overview of the questions that form sections 1 and 2.

Question	Overview	Response	Examples
1	Open question asking participants to describe what distinguishes the most/least severe outbursts	Words or short phrases given that characterise outbursts	Words such as ‘aggressive’, ‘shouting’, ‘hitting’
2–23	Participants asked to rate the frequency of 22 different behaviours	Three-point scale of never/rarely, sometimes, always/often	Behaviours include different types of aggression, self-injurious behaviour, talking to self
24–26, 28	Participants asked to rate duration, frequency, recovery time, and intensity of outbursts	Seven- or eight-point scale depending on the question	
27	How much eye contact is sought during outbursts	Three-point scale of less than baseline, baseline or more than baseline	

### Measures

The EOQ was developed for the wider study with consultation from focus groups and reference to the literature, and went through several stages of development, in the absence of another ‘gold standard’ measure. The EOQ is produced in full in the supplementary materials, and its development is described in detail elsewhere^10^. The SCQ is an autism screening tool, the results of which are not analysed in this study^22^.

### Statistical analyses

Statistical analyses comparing questions between least and most severe outbursts within the same individual were undertaken in R 4.2.2. Bivariate analysis was performed using McNemar-Bowker test as data were paired categorical data on a three-point scale^23^. Cohen’s G effect sizes and corresponding confidence intervals were produced for each question^21^. Questions comparing frequency, intensity, duration and recovery were initially assessed for normality using the Shapiro–Wilk test^24^. The data was found to be not normally distributed, and Wilcoxon signed-rank testing was performed as the data was on a large scale and therefore was analysed as if it were numerical data. Wilcoxon signed-rank testing compares two groups of non-parametric data^25^. To minimise type 1 errors that could occur from multiple McNemar-Bowker tests, Bonferroni correction was performed^26^.

The open question at the start of each section was analysed using content analysis^27^. Four participants were excluded from the content analysis as they did not complete both questions, leaving 210 responses. Categories were developed with definitions and examples given for each category (Appendix B). Every word and phrase given in answer to the question was assigned to at least one category. The first rater was author BS. A research assistant acted as the second rater and carried out coding on a randomly selected 25% of the responses to determine inter-rater reliability. Cohen’s Kappa was calculated for each category to account for agreement due to chance. Here, Kappa values below 0.60 indicates inadequate agreement, 0.60–0.79 moderate agreement, and 0.80–1 strong agreement^28^.

## Results

### Overall characteristics of emotional outbursts

**Figure 1:**
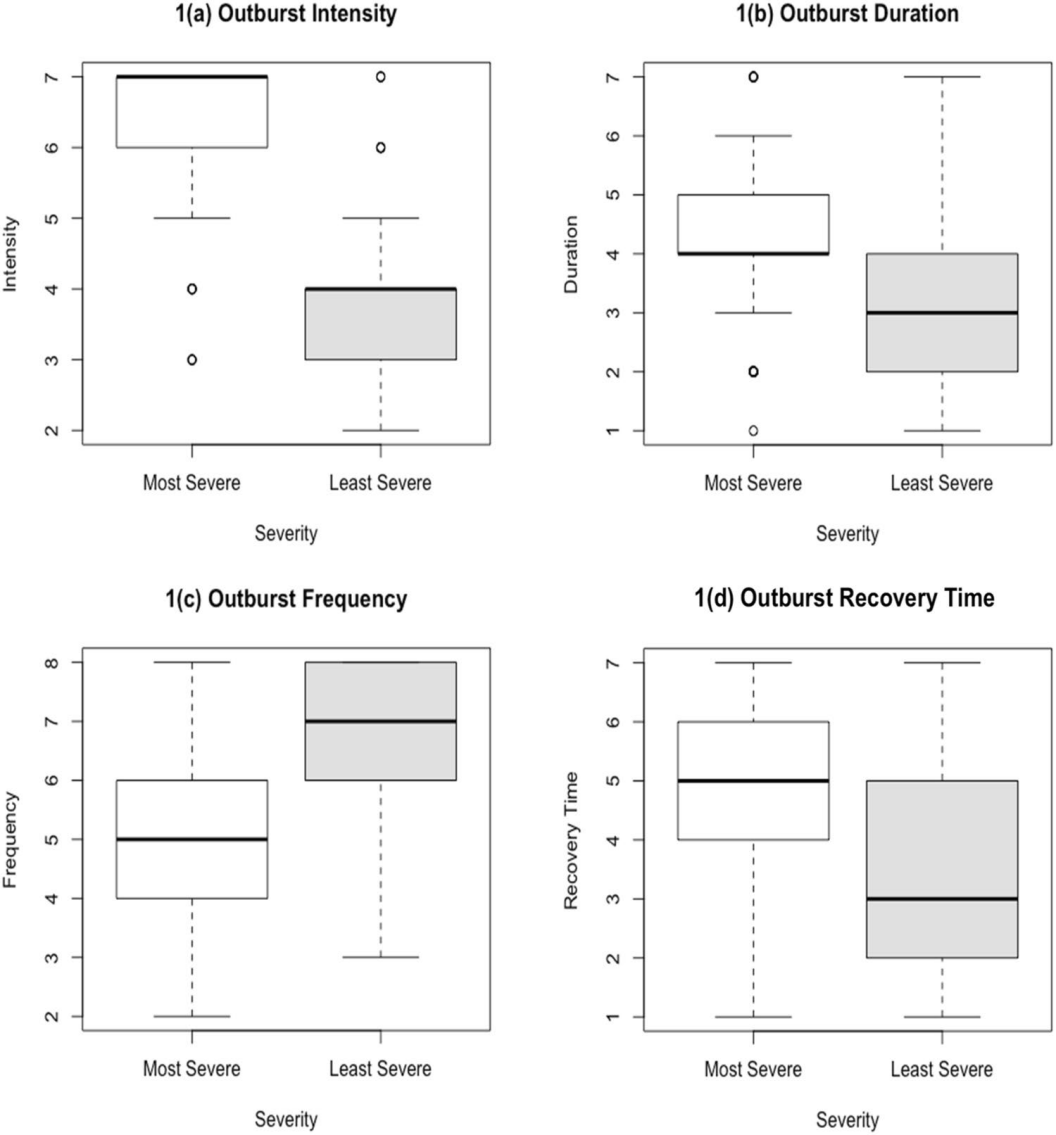
(**a**–**d**) Differences in duration, intensity, frequency and recovery time of most and least severe emotional outbursts. Duration is displayed on a scale of 1 (less than 5 min) and 7 (a day or more). Intensity is displayed on a scale of 1 (‘not at all angry or upset’) to 7 (‘as angry or upset as I have ever seen them’). Frequency is displayed on a scale of 1 (never occurs) to 8 (occurs more than once per day). Recovery is displayed on a scale of 1 (recovers in less than 5 min) to 7 (takes more than a day to recover). Full details on the scale are shown in the EOQ, found in the supplementary materials.

Figure 1a–d show the difference in characteristics of most and least severe outbursts generally. Most severe outbursts were noted to be longer in duration, be of a higher intensity and require a longer recovery time than least severe outbursts. However, it was reported that least severe outbursts occurred more frequently.

### Open question on outburst behaviours

Results from the content analysis of the open question are shown in Tables 2 and 3, displaying all behaviours for which interrater reliability was greater than 0.6 according to Cohen’s Kappa. Full results from the content analysis can be seen in Appendix C.

**Table 2:** Associations with most severe outbursts.

Category	Number of people reporting behaviour in open-ended questions (%)	Cohen’s Kappa for interrater reliability
Verbal aggression	181 (62.4)	0.841
Aggression towards property	96 (72.1)	0.829
Unspecified physical aggression	135 (86.5)	0.653
Self-injurious behaviours	68 (79.2)	0.877
Suicidal ideation expression	9 (100)	0.884
Duration ≥ 20 min	51 (94.4)	0.918

**Table 3:** Associations with least severe outbursts.

Category	Number of people reporting behaviour in open-ended questions (%)	Cohen’s Kappa for interrater reliability
Duration < 20 min	29 (78.4)	0.879
Behavioural indicative of emotion	109 (61.9)	0.747

### Effect sizes of each behaviour

**Figure 2:**
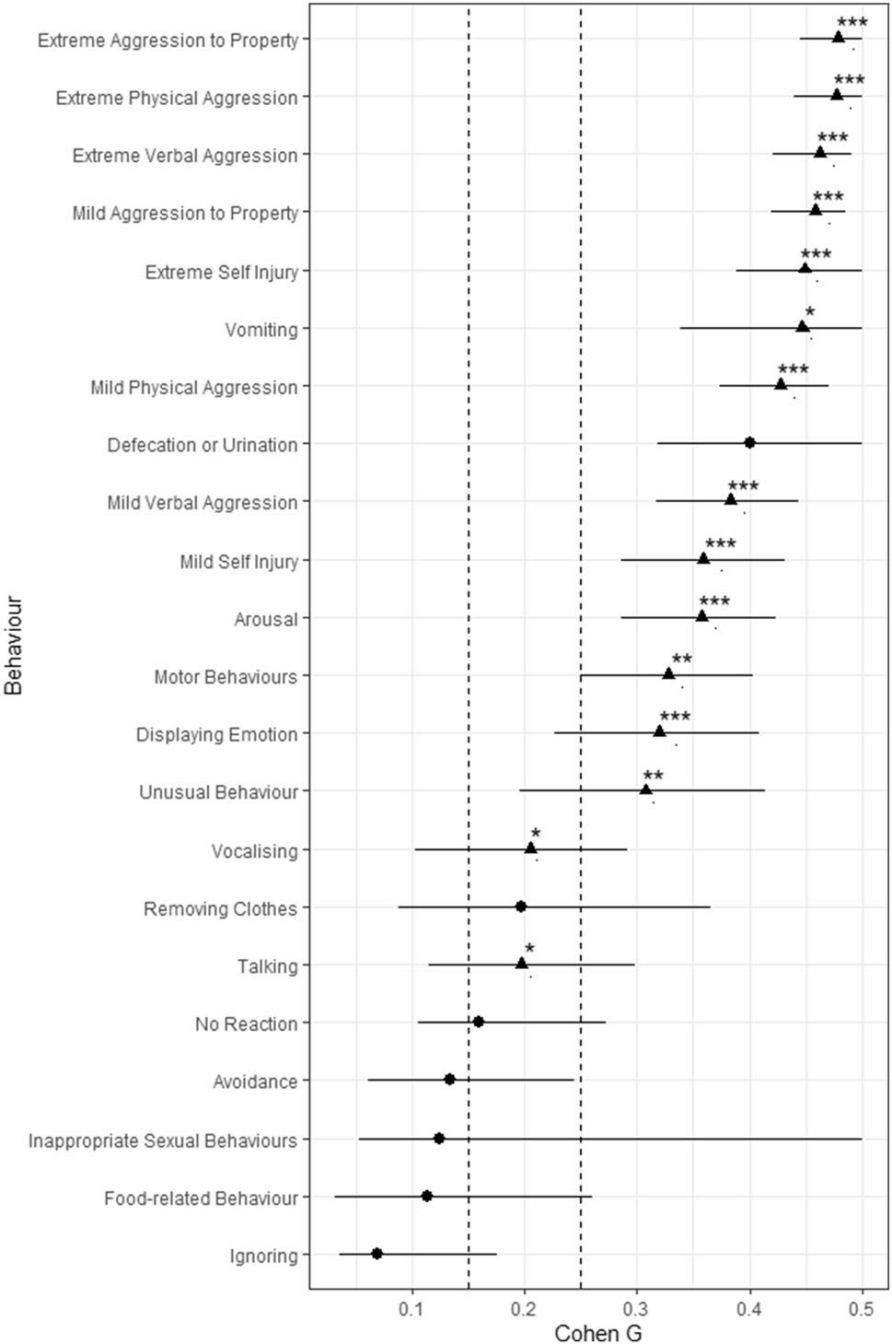
Association between individual behaviours and most severe outbursts. ***Indicates *p* < 0.001, **Indicates *p* < 0.01, *Indicates *p* < 0.05. Dotted line at 0.15 indicates boundary between small and medium effect size, dotted line at 0.25 indicates boundary between medium and large effect size^21^.

Figure 2 shows the results of the closed questions, displaying the association between a behaviour and most severe outbursts. It is clear from the table that all forms of aggression are strongly associated with most severe outbursts. Additionally, it was found that reduced eye contact was associated with most severe outbursts. No behaviours were found to be more associated with least severe outbursts than most severe outbursts.

Triangle denotes significant effect and circle indicates non-significant effect.

## Discussion

### Summary of findings

This was the first study to explore intra-individual differences in outburst characteristics in young people who experienced emotional outbursts at least once a month. The Emotional Outburst Questionnaire, a recently developed informant report measure, was used in this cross-sectional study of caregivers of individuals experiencing emotional outbursts.

From content analysis of open ended descriptions provided by caregivers, aggressive and self-injurious behaviours were generally noted as distinguishing features of most severe outbursts, whilst signs of emotion and complaining behaviours were common distinguishing features of least severe outbursts. Expression of suicidal ideation was identified as a distinguishing factor for most severe outbursts of some young people.

From responses to closed questions that asked caregivers to rate specific behaviours that may or may not be typical of the young person's outbursts, all forms of aggression were significantly associated with most severe outbursts. Moreover, some behaviours showed no significant difference when comparing most and least severe outbursts, such as avoidance behaviours, defecation and urination, and ignoring behaviours. Additionally, a decrease in eye contact compared to baseline was significantly associated with most severe outbursts.

It was found that most severe outbursts were reported as longer in duration, of a higher intensity and required longer recovery times than least severe outbursts, but least severe outbursts were reported to be more frequent.

Analysis of the open and closed question responses demonstrated convergent findings, with increased aggression of all forms, alongside self-injurious behaviours being more common in most severe outbursts. The five behaviours that showed no significant difference in frequency between most severe and least severe outbursts also showed no difference in their comparable categories in the content analysis (avoidance, defaecation/urination, food-related behaviours, ignoring, and no reaction to things around them). The question relating to inappropriate sexual behaviours could not be analysed due to results being heavily skewed towards never/rarely/not applicable, and no caregiver listed inappropriate sexual behaviours as a differentiator for either most severe or least severe outbursts.

Additionally, most severe outbursts were rated as being longer in duration in the answers of both open and closed questions and least severe outbursts found to be shorter in both sets of responses too.

The results of the present study support findings from previous literature characterising outbursts, with the most frequent behaviours shown in studies on individuals with PWS and ASD such as crying and forms of verbal aggression being noted frequently here (Fig. 2)^11,29^.

### Implications of findings

When considering behaviours associated with the most severe outbursts, comparisons can be drawn between the findings of the present study and previous literature on challenging behaviours. Here, all forms of aggression, including self-injurious behaviours were associated significantly with more severe outbursts. Earlier studies have categorised challenging behaviour as more severe if the behaviour causes major injury, or if physical intervention was required (e.g.^12^). The severity of an outburst therefore seems to be linked to the demands that constituent behaviours place on others. Whilst this is understandable given the informant report nature of the present work, it has important potential implications for future studies, such as using the behaviours found to be strongly associated with most severe outbursts to produce a short-form screening questionnaire. It has been noted previously that outbursts are often wrongly considered to be acute or resolving episodes, despite the evidence base showing they are chronic^4^. A short screening tool that could be used across a period of time to measure outbursts would therefore be useful. The ability to screen outbursts over time could prove useful when assessing high intensity but low frequeny outburst profiles, that otherwise would require lengthy evaluation periods during a trial. Moreover, profiling an individual’s outbursts could help to target interventions at specific types of outbursts or outburst behaviour, rather than for all outbursts they experience, and could help measure response to these interventions. Additionally, the present paper goes some way to answering the call from previous work to better define and characterise emotional outbursts^4^.

Aggressive behaviours appear to be a clear target for interventions, as various aggressive behaviours were strongly associated with most severe outbursts. Interventions for such behaviours exist, such as anger management strategies, though evidence of their effectiveness is inconclusive^30^.

Additionally, it was found that eye contact was generally at baseline in least severe outbursts, and generally less than baseline in most severe outbursts. This is consistent with previously reported differentiations of outburst intensity^31^. Gaze aversion in general appears to be enhanced in individuals with some neurodevelopmental disorders, including autism and fragile X syndrome^32,33^ so this finding must be evaluated in the context of a sample comprised of many young people with neurodevelopmental disorders. However, contextually, gaze aversion has been associated with managing an increased cognitive load on the individual at the time, pointing towards the particularly cognitively demanding nature of most severe outbursts^34^. In line with this, caregivers have reported that individuals experiencing outbursts can lose their usual capacities during the outburst^35^. This finding is also relevant to the measurement of outbursts because eye contact is easy to record objectively^34^, and so observation of this could contribute a less biased measurement of emotional outbursts for future intervention studies.

A further important finding of the present study was the presence of expressions of suicidal thoughts as a differentiator of most severe compared to least severe outbursts. Though this category only had nine responses endorsing it, all nine mentioned it in relation to most severe outbursts, highlighting the extent to which outbursts can impact wellbeing. Furthermore, expression of suicidal ideation was not included as part of the main questionnaire in the present study and is not generally included in questionnaires in previous work^6,11^. Individuals diagnosed with ASD show increased presentation of suicidal ideations and compared to the general population are significantly more likely to die by suicide^36,37^. There is a lack of literature explaining why this is the case, but the link between the expression of suicidal ideation and factors related to most severe emotional outbursts could be a point of focus for future research^38^. It is important to highlight that talking about suicide is not necessarily the same as having suicidal ideations, a limitation that is also present in studies on suicidal thoughts in individuals with an ASD diagnosis, but this does not detract from the importance of further study on this topic^39^.

The results of this study found that several behaviours are common across both most and least severe outbursts, such as displays of emotion and signs of verbal aggression. This could imply that most severe outbursts include least severe outbursts, and the behaviours that are common indicate building up of negative emotion. Outbursts have been described by those who experience them as emotional triggers, such as being ignored, misunderstood or being in environments that do not cater to their needs, that build up into displays of behaviour that feel uncontrollable such as aggression or self-injurious behaviour^40,41^.

### Limitations

Though this study has a relatively large sample size, some diagnoses were represented by a small number of individuals, so the generalisability of the results is restricted. Although some studies suggest outburst characteristics are similar across diagnoses, these studies generally have not included some of the least prevalent disorders in this study^5,6,11^.

Additionally, as with many studies on emotional outbursts, this study utilises caregiver reporting which may be subject to a number of biases. There are pragmatic difficulties as well as a different set of biases associated with self-report of emotional outbursts. But both caregiver and self-report are clearly important. Future research seeking more information on outbursts from the perspective of individuals showing them is much needed. In addition, more males were recruited than females to this study. This is an important limitation, as it has been found that males with intellectual disability show a higher prevalence of aggressive behaviours^42^. It is therefore possible that the imbalance between male and female participants could lead to the overstatement of aggressive behaviours in the present study.

Moreover, participants who stated that there was no variability in the severity of outbursts in the individual they cared for were excluded from this study, as they did not have responses for both sections 1 and 2 of the EOQ. From the original sample size of 268, 54 responders reported no variability in outburst severity. Here, there is a subset of participants that cannot be accounted for by the present findings but where possible should be accounted for in future work.

## Conclusion

In summary, the present study has shown using quantitative analyisis that there are behaviours that distinguish between most severe and least severe outbursts, including all forms of aggression, self-injury and arousal, all of which are associated with most severe outbursts. Eye contact was the only behaviour positively associated with least severe outbursts. Most severe outbursts were rated as being more intense, longer in duration, and requiring a longer recovery period, whilst least severe outbursts were found to be more frequent.

### Supplementary Information

Supplementary Information.

## Data Availability

The datasets generated and/or analysed during the current study are available in the Categorising Emotional Outbursts in Young People repository, https://osf.io/n8hgy/?view_only=e996af67c7d542e694d0664858381875.
