# Supplementary material for: Characterising the behaviours in most severe and least severe emotional outbursts in young people
Source: Sci Rep. 2024 Feb 5;14:2957. doi: 10.1038/s41598-024-52732-x (PMC10844367; doi:10.1038/s41598-024-52732-x)
Supplement: Supplementary file 1 — Supplementary Information. [file 41598_2024_52732_MOESM1_ESM.pdf]

Supplementary Materials

Appendix A

Diagnoses and frequencies of the individuals' participants cared for.

Diagnosis	Frequency	Percentage
Anxiety	75	35.0
Learning Disability	57	26.6
Attention Deficit Hyperactivity Disorder	51	23.8
Diagnosis Under Assessment	25	11.7
Learning Difficulty	23	10.7
Sensory Disorder	23	10.7
Attachment Disorder	16	7.5
Depression	13	6.1
No Diagnosis	12	5.6
Cornelia deLange Syndrome	12	5.6
Foetal Alcohol Disorder	11	5.1
Deafness	9	4.2
Prader-Willi Syndrome	9	4.2
Noonan syndrome	8	3.7
William's syndrome	8	3.7
Hypermobility	7	3.3
Pathological Demand Avoidance	7	3.3
Disruptive Behaviour Disorder	7	3.3
Other Genetic	6	2.8
CHARGE syndrome	5	2.3
Tuberous sclerosis complex	5	2.3

The full list of diagnoses and frequencies can be found in Appendix A.

Percentage adds up to more than 100 as some participants listed multiple diagnoses.

Appendix B

Content analysis categories and definitions/examples

Category	Definition/Examples
Aggression towards others	E.g., biting, hitting, includes aggressive gestures towards others, including use of weapons
Aggression towards property	E.g., throwing objects, breaking things
Arguing	Including disagreement, answering back
Attempts at de-escalation	E.g., attempting to calm down or regain control
Behaviour indicative of emotion	E.g., crying, descriptions of facial expressions are signs of emotions
Being inflexible	E.g., fussy, outburst occurs if individual is asked to a less preferred activity
Bodily responses	Unintentional bodily responses such as vomiting, or inducing a headache or tiredness
Caregiver label of anger	Including anger, frustrated, rage
Caregiver label of anxiety	Including anicky, agitated
Caregiver label of distress	Including emotional, sad, upset
Caregiver label of confusion	E.g., confused, irrational thinking
Contextually inappropriate behaviours	E.g., inappropriate laughing, withdrawal into roleplay
Defiance/refusal	Including stubborn behaviours
Demanding	E.g., follows when walk away/demanding of attention/asking or pleading
Difficult to resolve	E.g., can't reason with the person
Dramatic behaviours	E.g., catastrophising
Drug use	E.g., alcohol or illicit drug use during outburst
Duration of 20 mins or longer	Duration of 20 mins or longer, or expression of extended/long duration
Duration of less than 20 mins	Duration of less than 20 mins, or expression of short duration
Easier to resolve	E.g., resolved by caregiver walking away, or caregiver saying that outbursts are easy to resolve
Easily triggered	Events perceived to be insignificant trigger the outburst
Escape behaviours	E.g., running off or dropping to the floor
Expression of remorse during or after an outburst	E.g., frequent apologies after the outburst
Expressions of suicidal ideation	Vocalising suicidal ideations or threatening suicide
External triggers	Outburst triggered by external factors
Feels misunderstood	E.g., people not understanding the individual during outburst or related to trigger
Increased motor activity	E.g., hyperactive behaviour, stomping, motor tic behaviour
Increased non-word sounds	Such as non-word moaning or groaning, but not including aggressive sounds such as screaming
Increased talking	Increased activity that is verbal communication
Intentional antisocial behaviours	E.g., intentional urination/defecation/spitting, can include other anti-social behaviours such as arguments in public
Long lasting effects	E.g., being in pain post-outburst
Lying	Statements that aren't true
Negative emotional impact on carer	Carer expressing feelings of hopelessness/sadness/fear
Obsessional behaviours or thoughts	Obsessional or compulsive behaviours occurring during outburst
Occurs frequently	Frequency of at least once a week

Category	Definition/Examples
Occurs rarely	Frequency of less than once a week
Perceived manipulation	Caregiver perceiving individual's behaviour to be controlling or manipulative
Perceived meanness	E.g., rudeness, label of intentional mean behaviour
Physiological arousal	Such as red face, dribbling, tensed up
Repetitive behaviours	E.g., repetitive speech or movements
Seeks support from caregiver	E.g., seeking affection, seeking physical pressure, seeking reassurance including visual reassurance
Self-deprecating behaviour	Verbal self-deprecation from the individual, saying they don't deserve things
Self-injurious behaviours	Actions of harm towards self, such as banging head
Sensory seeking	Specific description of sensory seeking behaviour from the individual during the outburst
Shutting down behaviour	Not verbally communicating when the individual is normally verbally communicative or avoiding physical contact
The individual has no control/awareness during the outburst	E.g., no control, lack of insight, meltdown, also including no awareness of surroundings
Triggered by bodily state indicators of low resources	E.g., tiredness, low blood sugar as triggers
Triggered by fear	E.g., fear of a new situation
Uncharacteristic behaviour	Behaviour shown is not normal/is uncharacteristic for the individual
Unexpected outburst	E.g., outburst being unexpected or sudden
Unspecified physical aggression	Statements of aggression not clarified by the caregiver
Verbal aggression	Verbalising threats, shouting, swearing, includes non-word aggressive vocalisation such as screaming
Whining/complaining	E.g., whining or complaining

Appendix C

Results of the content analysis in full

Category description	Frequency for MS	Frequency for LS
Aggression towards others	55	10
Aggression towards property	96	37
Arguing	0	13
Attempts at de-escalation	1	6
Behaviour indicative of emotion	67	109
Contextually inappropriate behaviours	2	9
Being inflexible	0	3
Bodily responses	6	4
Caregiver label of anger	27	30
Caregiver label of anxiety	11	10
Caregiver label of distress	14	10
Caregiver label of confusion	5	1
Defiance/refusal	6	24
Demanding	5	8
Difficult to resolve	36	7
Dramatic behaviours	1	1
Drug use	1	0
Duration of 20 mins or longer	51	3
Duration of less than 20 mins	8	29
Easier to resolve	2	13
Easily triggered	2	4
Escape behaviours	32	27
Expression of remorse during or after an outburst	1	2
Expressions of suicidal ideation	9	0
External triggers	0	2
Feels misunderstood	1	1
Increased motor activity	28	35
Increased non-word sounds	2	14
Increased talking	5	17
Intentional antisocial behaviours	2	1
Long lasting effects	1	2
Lying	0	1
Negative emotional impact on carer	6	1
Obsessional behaviours or thoughts	0	3
Occurs frequently	3	3
Occurs rarely	0	0
Perceived manipulation	11	7

Category description	Frequency for MS	Frequency for LS
Perceived meanness	6	8
Physiological arousal	14	32
Repetitive behaviours	15	28
Seeks support from caregiver	1	10
Self-deprecating behaviour	4	6
Self-injurious behaviours	68	18
Sensory seeking	0	1
Shutting down behaviour	13	36
The individual has no control/awareness during the outburst	31	6
Triggered by bodily state indicators of low resources	0	2
Triggered by fear	0	1
Uncharacteristic behaviour	2	1
Unexpected outburst	4	1
Unspecified physical aggression	135	21
Verbal aggression	181	109
Whining/complaining	6	29

The Emotional Outburst Questionnaire

The term "*emotional outburst*" refers to a highly emotional or explosive episode, where at least one of the behaviours listed below (items 2-23) is displayed. Emotional outbursts may also be known as "*meltdowns*", "*crisis*", "*behavioural breakdown", "blips*", "*rages*", "*temper outbursts*", "*tantrums*", or "*tempers*".

In this questionnaire, we want you to think about the **most severe** and **least severe** emotional outbursts **within the past month** that the individual you care for has displayed and the characteristics associated with each type of emotional outburst, such as behaviours, frequency, and duration. In terms of the **severity** of emotional outbursts, we are referring to how disruptive and negatively impactful they are to the person and/or those around them **at the time** of the emotional outburst.

If you feel that the **severity** of emotional outbursts is always the same, please answer the questions relating to the **most severe** type. We recognise that some questions may be difficult to answer, as emotional outbursts can vary greatly depending on the context. However, please try to give an average for these questions, as this will help us to better understand emotional outbursts over a range of contexts.

First, we would like you to consider the **most severe** emotional outbursts that the individual you care for has displayed **within the past month**.

1	Please list **up to 20 words** to describe what distinguishes the **most severe** emotional outbursts. *(e.g. physically aggressive, screaming, at least an hour)*	

Please indicate your answer for each of the following items by ticking the appropriate box (☐).

During the **most severe** emotional outbursts, how often does the individual you care for display the following behaviours?	Not applicable/never/rarely (0-3 times out of 10 outbursts)	Sometimes (4-6 times out of 10 outbursts)	Often/always (7-10 times out of 10 outbursts)
2 Behavioural indicators of emotion *(e.g. angry or annoyed facial expressions, crying, signs of distress, whining)*	☐	☐	☐
3 Mild verbal aggression *(e.g. insults, name-calling, screaming, shouting, swearing)*	☐	☐	☐
4 Extreme verbal aggression *(e.g. threats of violence)*	☐	☐	☐

5	Non-speech vocalisations (*e.g. making sounds or noises*)	☐	☐	☐
6	Mild aggression towards property *(e.g. defacing walls, ripping clothing, slamming door, throwing objects down)*	☐	☐	☐
7	Extreme aggression towards property *(e.g. breaking objects, smashing windows, throwing objects dangerously)*	☐	☐	☐
		Not applicable/never/rarely (0-3 times out of 10 outbursts)	Sometimes (4-6 times out of 10 outbursts)	Often/always (7-10 times out of 10 outbursts)
8	Mild physical aggression towards others **without physical injury** *(e.g. biting, grabbing, hitting, kicking, pulling hair, pushing, scratching, spitting, throwing objects at people)*	☐	☐	☐
9	Extreme physical aggression towards others **with physical injury** *(e.g. biting, grabbing, hitting, kicking, pulling hair, pushing, scratching, throwing objects at people)*	☐	☐	☐
10	Mild self-injurious behaviours **without serious injury** (no cuts, bruises, burns, etc) *(e.g. banging head, biting, hitting self, hitting wall, holding breath, picking skin, pulling hair)*	☐	☐	☐
11	Extreme self-injurious behaviours **with serious injury** *(e.g. banging head, biting, hitting self, hitting wall, picking skin, picking rectum, pulling hair)*	☐	☐	☐
		Not applicable/never/rarely (0-3 times out of 10 outbursts)	Sometimes (4-6 times out of 10 outbursts)	Often/always (7-10 times out of 10 outbursts)

12	Talking to self & others<br>*(e.g. agitated talking, repetitive speech, self-deprecating speech)*	☐	☐	☐
13	Increased motor activity<br>*(e.g. flailing arms, non-directed kicking, pacing, repetitive behaviours, rushing about, stamping feet, tics)*	☐	☐	☐
14	Increased physiological arousal<br>*(e.g. red face, salivating, sweating)*	☐	☐	☐
15	Avoidance<br>*(e.g. dropping to floor, going to room, leaving situation, running away)*	☐	☐	☐
16	Removing items of clothing	☐	☐	☐
17	Defecation or urination	☐	☐	☐
18	Contextually inappropriate sexual behaviours	☐	☐	☐
19	Ignoring or not talking to certain people	☐	☐	☐
20	Not reacting to things going on around them	☐	☐	☐
		Not applicable/never/rarely (0-3 times out of 10 outbursts)	Sometimes (4-6 times out of 10 outbursts)	Often/always (7-10 times out of 10 outbursts)
21	Food-related behaviours<br>(*e.g. grabbing, pleading for, seeking, or stealing food*)	☐	☐	☐
22	Making themselves sick<br>(*e.g. retching or vomiting*)	☐	☐	☐
23	Unusual behaviours	☐	☐	☐

Advice and support around protecting the individual you care for is available via the NSPCC, Papyrus, or Samaritans.

Website: nspcc.org.uk
Email: help@nspcc.org.uk
Telephone: 0808 800 5000

Website: papyrus-uk.org
Email: pat@papyrus-uk.org
Telephone: 0800 068 4141

Website: samaritans.org
Email: jo@samaritans.org
Telephone: 116 123

24	How often do the **most severe** emotional outbursts occur?	☐ Never	☐ Less than once a month	☐ Once a month	☐ 2-3 times a month	☐ Once a week	☐ 2-3 times a week	☐ Once a day	☐ More than once a day
25	How long do the **most severe** emotional outbursts last?	☐ Less than 5 minutes	☐ 5-15 minutes	☐ 15-30 minutes	☐ 30 minutes to 1 hour	☐ 1-2 hours	☐ 2 hours to a day	☐ A day or more	
26	How angry or upset does the person get during the **most severe** emotional outbursts?	☐ 1 Not angry or upset at all	☐ 2	☐ 3	☐ 4	☐ 5	☐ 6	☐ 7 As angry or upset as I have ever seen them	
27	Compared to baseline behaviour, how much eye contact does the person seek from you during the **most severe** emotional outbursts?	☐ Less than baseline	☐ Same as baseline	☐ More than baseline					
28	How long does it take for the person to recover from the **most severe** emotional outbursts (i.e. from the end of emotional outburst behaviours to when behaviour is back to normal)?	☐ Less than 5 minutes	☐ 5-15 minutes	☐ 15-30 minutes	☐ 30 minutes to 1 hour	☐ 1-2 hours	☐ 2 hours to a day	☐ A day or more	

Now, we would like you to consider the **least severe** emotional outbursts that the individual you care for has displayed **within the past month**, that nevertheless disrupt and negatively impact them and/or those around them. We are referring to episodes that are different from the person's normal or baseline behaviour. The term "*emotional outburst*" refers to a highly emotional or explosive

episode, where at least one of the behaviours listed above (items 2-23) is displayed. Emotional outbursts may also be known as "*meltdowns*", "*crisis*", "*behavioural breakdown", "blips*", "*rages*", "*temper outbursts*", "*tantrums*", or "*tempers*".

If you feel that the **severity** of emotional outbursts is always the same, please tick the 'Not applicable' box below and continue from item 57.

29	Please list **up to 20 words** to describe what distinguishes the **least severe** emotional outbursts.<br>*(e.g. crying, red face, no more than 5 minutes)*	☐ Not applicable

Please indicate your answer for each of the following items by ticking the appropriate box (☐).

	During the **least severe** emotional outbursts, how often do they display the following behaviours?	Not applicable/never/rarely (0-3 times out of 10 outbursts)	Sometimes (4-6 times out of 10 outbursts)	Often/always (7-10 times out of 10 outbursts)
30	Behavioural indicators of emotion *(e.g. angry or annoyed facial expressions, crying, signs of distress, whining)*	☐	☐	☐
31	Mild verbal aggression *(e.g. insults, name-calling, screaming, shouting, swearing)*	☐	☐	☐
32	Extreme verbal aggression *(e.g. threats of violence)*	☐	☐	☐
33	Non-speech vocalisations (*e.g. making sounds or noises*)	☐	☐	☐
34	Mild aggression towards property *(e.g. defacing walls, ripping clothing, slamming door, throwing objects down)*	☐	☐	☐
35	Extreme aggression towards property *(e.g. breaking objects, smashing windows, throwing objects dangerously)*	☐	☐	☐
36	Mild physical aggression towards others **without physical injury** *(e.g. biting, grabbing, hitting, kicking,*	☐	☐	☐

	pulling hair, pushing, scratching, spitting, throwing objects at people)			
		Not applicable/never/rarely (0-3 times out of 10 outbursts)	Sometimes (4-6 times out of 10 outbursts)	Often/always (7-10 times out of 10 outbursts)
37	Extreme physical aggression towards others **with physical injury** *(e.g. biting, grabbing, hitting, kicking, pulling hair, pushing, scratching, throwing objects at people)*	☐	☐	☐
38	Mild self-injurious behaviours **without serious injury** (no cuts, bruises, burns, etc) *(e.g. banging head, biting, hitting self, hitting wall, holding breath, picking skin, pulling hair)*	☐	☐	☐
39	Extreme self-injurious behaviours **with serious injury** *(e.g. banging head, biting, hitting self, hitting wall, picking skin, picking rectum, pulling hair)*	☐	☐	☐
40	Talking to self & others *(e.g. agitated talking, repetitive speech, self-deprecating speech)*	☐	☐	☐
41	Increased motor activity *(e.g. flailing arms, non-directed kicking, pacing, repetitive behaviours, rushing about, stamping feet, tics)*	☐	☐	☐
42	Increased physiological arousal *(e.g. red face, salivating, sweating)*	☐	☐	☐
43	Avoidance *(e.g. dropping to floor, going to room, leaving situation, running away)*	☐	☐	☐
		Not applicable/never/rarely (0-3 times out of 10 outbursts)	Sometimes (4-6 times out of 10 outbursts)	Often/always (7-10 times out of 10 outbursts)
44	Removing items of clothing	☐	☐	☐

45	Defecation or urination	☐	☐	☐
46	Contextually inappropriate sexual behaviours	☐	☐	☐
47	Ignoring or not talking to certain people	☐	☐	☐
48	Not reacting to things going on around them	☐	☐	☐
49	Food-related behaviours (*e.g. grabbing, pleading for, seeking, or stealing food*)	☐	☐	☐
		Not applicable/never/rarely (0-3 times out of 10 outbursts)	Sometimes (4-6 times out of 10 outbursts)	Often/always (7-10 times out of 10 outbursts)
50	Making themselves sick (*e.g. retching or vomiting*)	☐	☐	☐
51	Unusual behaviours	☐	☐	☐

Advice and support around protecting the individual you care for is available via the NSPCC, Papyrus, or Samaritans.

Website: nspcc.org.uk
Email: help@nspcc.org.uk
Telephone: 0808 800 5000

Website: papyrus-uk.org
Email: pat@papyrus-uk.org
Telephone: 0800 068 4141

Website: samaritans.org
Email: jo@samaritans.org
Telephone: 116 123

52	How often do the **least severe** emotional outbursts occur?	☐ Never	☐ Less than once a month	☐ Once a month	☐ 2-3 times a month	☐ Once a week	☐ 2-3 times a week	☐ Once a day	☐ More than once a day

53	How long do the **least severe** emotional outbursts last?	☐ Less than 5	☐ 5-15 minutes	☐ 15-30 minutes	☐ 30 minut	☐ 1-2 hours	☐ 2 hours	☐ A day or more

		minut es			es to 1 hour		to a day	
54	How angry or upset does person get during the **least severe** emotional outbursts?	☐ 1 Not angry or upset at all	☐ 2	☐ 3	☐ 4	☐ 5	☐ 6	☐ 7 As angry or upset as I have ever seen them
55	Compared to baseline behaviour, how much eye contact does the person seek from you during the **least severe** emotional outbursts?	☐ Less than baseline			☐ Same as baseline		☐ More than baseline	
56	How long does it take for person to recover from the **least severe** emotional outbursts (i.e. from the end of emotional outburst behaviours to when behaviour is back to normal)?	☐ Less than 5 minut es	☐ 5-15 minut es	☐ 15-30 minut es	☐ 30 minut es to 1 hour	☐ 1-2 hours	☐ 2 hours to a day	☐ A day or more

We would like you to consider **in general, all emotional outbursts** the individual you care for has displayed **within the past month**.

Please indicate your answer for each item by ticking the appropriate box (☐).

57	How often do emotional outbursts occur?	☐ Never	☐ Less than once a month	☐ Once a month	☐ 2-3 times a month	☐ Once a week	☐ 2-3 times a week	☐ Once a day	☐ More than once a day

	When the individual you care for is in the following places, how often do emotional outbursts occur?	Not applicable/never/rarely (0-3 times out of 10)	Sometimes (4-6 times out of 10)	Often/always (7-10 times out of 10)
58	A place that makes them feel safe	☐	☐	☐
59	A place that makes them feel unsafe	☐	☐	☐
60	A place that they are familiar with *(e.g. at a relative/friend's house)*	☐	☐	☐
61	A place that they are unfamiliar with *(e.g. whilst on holiday away from home)*	☐	☐	☐
62	A place that they feel is private *(e.g. in their room)*	☐	☐	☐
63	A place that they feel is public *(e.g. at a shop)*	☐	☐	☐

	When the individual you care for is with the following people, how often do emotional outbursts occur?	Not applicable/never/rarely (0-3 times out of 10)	Sometimes (4-6 times out of 10)	Often/always (7-10 times out of 10)
64	Someone that makes them feel safe (*e.g. a parent/caregiver)*	☐	☐	☐
65	Someone that makes them feel unsafe *(e.g. a dentist)*	☐	☐	☐
66	Someone familiar *(e.g. a teacher)*	☐	☐	☐
67	Someone unfamiliar *(e.g. a cashier at a shop)*	☐	☐	☐

68	Someone they like	☐	☐	☐
69	Someone they dislike	☐	☐	☐
70	Someone they are jealous of	☐	☐	☐

When the individual you care for is in the following states, how often do emotional outbursts occur?		Not applicable/never/rarely (0-3 times out of 10)	Sometimes (4-6 times out of 10)	Often/always (7-10 times out of 10)
71	Tired	☐	☐	☐
72	Hungry or thirsty	☐	☐	☐
73	Consumed too much of one type of food or drink *(e.g. caffeine)*	☐	☐	☐
74	Illness	☐	☐	☐
75	In pain	☐	☐	☐
76	In a bad mood or having a bad day	☐	☐	☐

When the following trigger events occur, how often do they lead to an emotional outburst?		Not applicable/never/rarely (0-3 times out of 10)	Sometimes (4-6 times out of 10)	Often/always (7-10 times out of 10)
77	Planned transition from one activity to another	☐	☐	☐
78	Change in own routine	☐	☐	☐
79	Change in another's routine	☐	☐	☐
80	Change in expectation	☐	☐	☐
81	Being fixated on a thought or idea	☐	☐	☐
82	Specific phobia or fear	☐	☐	☐
83	Food-related triggers	☐	☐	☐
84	Concerns for own property (e.g. losing something or worried about losing something)	☐	☐	☐

		Not applicable/never/rarely (0-3 times out of 10)	Sometimes (4-6 times out of 10)	Often/always (7-10 times out of 10)
85	Not being given or not being able to do something the person wants	☐	☐	☐
86	Having to wait before being given or being able to do something	☐	☐	☐
87	Being asked to do something the person may or may not want to do	☐	☐	☐
88	Doing a boring task	☐	☐	☐
89	Doing a difficult task	☐	☐	☐
90	Doing a repetitive task	☐	☐	☐
		Not applicable/never/rarely (0-3 times out of 10)	Sometimes (4-6 times out of 10)	Often/always (7-10 times out of 10)
91	Doing a new task	☐	☐	☐
92	Under time pressure (e.g. getting ready in the morning)	☐	☐	☐
93	Disagreement with others	☐	☐	☐
94	Being told off, criticised, or accused of making a mistake	☐	☐	☐
95	Being teased	☐	☐	☐
96	Being apart from parent(s)/caregiver	☐	☐	☐
97	Not receiving enough attention or being ignored	☐	☐	☐
98	Receiving too much attention	☐	☐	☐
99	Feeling of being treated unfairly	☐	☐	☐
100	Someone not understanding the individual you care for	☐	☐	☐
101	The individual you care for not understanding someone else	☐	☐	☐
		Not applicable/never/ra	Sometimes (4-6 times out of 10)	Often/always (7-10 times out of 10)

		rely<br>(0-3 times out of 10)		
10<br>2	Not understanding what is going on	☐	☐	☐
10<br>3	Receiving conflicting information	☐	☐	☐
10<br>4	Light is too bright	☐	☐	☐
10<br>5	Sudden or loud noises	☐	☐	☐
10<br>6	Temperature is too hot or too cold	☐	☐	☐
10<br>7	Particular smells or strong smells	☐	☐	☐
10<br>8	Touch-related over-sensitivity (e.g. uncomfortable seat or sudden touch)	☐	☐	☐
10<br>9	Other sensory-related triggers<br>Specify:	☐	☐	☐
11<br>0	Medication side-effect	☐	☐	☐
11<br>1	Mood of parent/caregiver	☐	☐	☐
11<br>2	No reason/out of the blue	☐	☐	☐
11<br>3	How confident are you in your answers above (items 77-112) relating to the triggers that lead to emotional outbursts?	Not confident<br>☐	Quite confident<br>☐	Very confident<br>☐

How successful are the following management strategies in calming emotional outbursts of the individual you care for?		Not applicable/never/ra rely<br>(0-3 times out of 10)	Sometimes<br>(4-6 times out of 10)	Often/always<br>(7-10 times out of 10)
114	Physical or verbal comfort	☐	☐	☐
115	Discussion or persuasion	☐	☐	☐

116	Calming or relaxation strategies	☐	☐	☐
117	Giving them what they want	☐	☐	☐
118	Visual aids	☐	☐	☐
119	Punishment or threat of punishment	☐	☐	☐
120	Negotiation	☐	☐	☐
121	Actively ignoring behaviour	☐	☐	☐
122	Moving them or others from situation	☐	☐	☐
123	Distraction	☐	☐	☐
124	Showing empathy	☐	☐	☐

How often does the individual you care for display the following behaviours **after** emotional outbursts?		Not applicable/never/rarely (0-3 times out of 10 outbursts)	Sometimes (4-6 times out of 10 outbursts)	Often/always (7-10 times out of 10 outbursts)
125	Apologising	☐	☐	☐
126	Blaming others	☐	☐	☐
127	Seeking reassurance or comfort	☐	☐	☐
128	Appearing withdrawn	☐	☐	☐
129	Staying in a bad mood	☐	☐	☐
130	Feeling anxious	☐	☐	☐
131	Feeling sad	☐	☐	☐
132	Behaving as if nothing had happened	☐	☐	☐

		Never/rarely (0-3 times out of 10 outbursts)	Sometimes (4-6 times out of 10 outbursts)	Often/always (7-10 times out of 10 outbursts)
133	How often are you there to witness the emotional outbursts when they occur?	☐	☐	☐
